# Assessment of veterinary pharmaceutical warehouse management practices and its associated challenges in four selected zones and Bahir Dar city of Amhara regional state, Ethiopia

**DOI:** 10.3389/fvets.2024.1336660

**Published:** 2024-05-07

**Authors:** Abibo Wondie Mekonen, Tadilo Sintayehu, Alem Endeshaw Woldeyohanins, Yesuneh Tefera Mekasha, Berhanemeskel Weldegerima Atsbeha

**Affiliations:** ^1^Department of Veterinary Pharmacy, Pharmaceutical Supply Chain Management, University of Gondar, Gondar, Ethiopia; ^2^Department of Logistic and Supply Chain Management, University of Gondar, Gondar, Ethiopia; ^3^Department of Social and Administrative Pharmacy, School of Pharmacy, College of Medicine and Health Sciences, University of Gondar, Gondar, Ethio; ^4^Department of Veterinary Pharmacy, Pharmaceutical Quality Assurance and Regulatory Affairs, University of Gondar, Gondar, Ethiopia

**Keywords:** veterinary pharmaceuticals, veterinary clinics, veterinary drug wholesalers, warehouse management practices, Amhara region, Ethiopia

## Abstract

A pharmaceutical warehouse is part of the pharmaceutical supply chain and is essential to maintaining the quality and efficacy of veterinary pharmaceuticals for successful animal health service delivery. However, poor storage conditions, improper handling, and inappropriate use and disposal constitute challenges for veterinary supplies in animal health services. Therefore, this study aimed to assess the existing practices and challenges in warehouse management in government veterinary clinics and private veterinary drug wholesalers in Ethiopia. A cross-sectional study was conducted on 37 veterinary health facilities in four selected zones (south Gondar, west Gondar, central Gondar, and west Gojam zones) and Bahir Dar administrative city. Zones were selected using a simple random sampling technique. Data was collected using a structured questionnaire, pre-defined and tested observational checklists, and semi-structured interview guides. Descriptive statistics were used to analyze the quantitative data, while qualitative data was analyzed using a thematic approach. The study revealed the presence of poor stock management practices, such as the absence of standard operating procedures for warehouse activities in ~59.5% of facilities surveyed. In none of the surveyed facilities, bin cards and system software utilization were satisfactory. The absence of disposal guidelines was detected in 83.8% of the facilities, and the practice of timely disposal of expired drugs was not satisfactory. Compared to the government veterinary clinics, private veterinary drug wholesalers had better storage practices (86.25%) following theoretical recommendations. The storage conditions in government clinics were rated poor at 48.3% (>80%, which is the limit to the acceptable rate for good storage conditions). The challenges of inadequate infrastructure, a lack of qualified staff, problems with the availability and affordability of pharmaceutical products, insufficient regulatory practice, and budget constraints were identified. A holistic approach involving related stakeholders should be followed to improve the existing challenges and the sector's efficiency.

## Introduction

In Africa, livestock plays a significant role in economic growth, and Ethiopia has the biggest livestock population on the continent (1). However, Ethiopia's livestock productivity is lower compared to the country's livestock population (2, 3). The improper handling, inappropriate use, and poor disposal practices of veterinary pharmaceuticals are global issues that reduce livestock productivity and affect successful animal health services (4–7).

Veterinary pharmaceuticals are the foundation of animal disease diagnosis, treatment, prevention, and control, while the quality, safety, and efficacy of pharmaceuticals in a supply chain significantly depend on warehouse management (8, 9). A warehouse is a functional and organizational structure designed for storing tangible products (stocks) in a dedicated area using proven technology, managed by a group of people, and furnished with the necessary technical tools. Warehouse management (WM) is part of a logistics system that comprises accepting, storing, issuing, recording, and tracking information flow throughout the process. In pharmaceutical supply chain management (SCM), strategic warehouse operations are the foundation of a service's success (10, 11).

Good veterinary supplies management practices play a pivotal role in maintaining the quality of drugs and ensuring the delivery of successful animal health services. They also reduce non-conformities, promote efficient labor allocation, and reduce average operation time. Studies indicate that effective WM practices can save 15.7% of space across manufacturing and healthcare organizations and account for 2% to 5% of the cost of sales (12, 13). On the other hand, improper handling of veterinary drugs impedes the quality of animal health services; it is also a leading cause of the risk of antimicrobial resistance (AMR) in humans (14).

According to the European Medicine Association (EMA), to achieve good veterinary drug management practices and reduce the wastage of veterinary supplies (VS) at any veterinary service delivery point (SDP), proper installation of warehouse and storage facilities, trained and qualified professionals, and standardized written manuals, policies, and guidelines are necessary components that must be fulfilled (15). In high-income nations, systems have been established to evaluate and track the quality of veterinary pharmaceuticals (VPs) available in the market and at SDP. However, most low- and middle-income nations find it difficult to monitor the use of veterinary drugs in livestock (4).

In most African countries, the poor advancement of animal healthcare is due to the influx of poor-quality drugs and complex drug distribution chains that involve many actors (both formal and informal) (4, 16, 17). According to a study conducted in Sub-Saharan Africa, the weak distribution of infrastructure and services and the multiplication of non-professional actors in the veterinary drug chains are the major constraints for farmers to access good-quality drugs (18). Another study conducted in Dares Salaam, Tanzania, revealed that poor record-keeping and the lack of guidelines on the appropriate disposal of veterinary medicines are the factors that affect drug handling and management (19). A study conducted in Nigeria showed that Africa had lost USD 4 billion due to preventable livestock diseases (4).

Until the French Veterinary Mission started offering modern veterinary services in 1908, Ethiopia's veterinary healthcare services were managed traditionally (20). However, currently, the government provides considerable veterinary healthcare services through clinics in every district and kebele (Ethiopia's fourth administrative level, following regions, zones, and districts). Veterinary clinics, designated to deliver veterinary healthcare services, veterinary pharmacy importers, wholesalers, and retail outlets, the majority of which are franchise veterinary drug businesses, professionally distribute veterinary pharmaceuticals throughout the country (2). Due to the interdependence and dependence among the activities in the veterinary pharmaceutical supply chain, a failure in one activity will have a detrimental impact on subsequent actions. For the provision of high-quality, sustainable animal health services, various service delivery institutions, such as veterinary drug importers, wholesalers, retailers, governmental animal health service providers (AHSP), animal health administrators, research and educational institutions, policymakers, legal affairs, and livestock owners, must all work together in the veterinary drug supply chain (20–22).

Literature indicates that the livestock sub-sector in Ethiopia is vulnerable to several veterinary pharmaceutical supply chain issues, including poor drug handling during purchase, irrational use of drugs, illegal marketing, poor-quality medicines, a lack of waste management practices, low rates of adherence to rules and policies, a lack of qualified public and private animal health services, and a lack of qualified and trained staff (5, 6, 21, 23, 24). Another study conducted in Ethiopia on the quality of veterinary drugs during post-marketing surveillance, re-registration, consignment checking, and pre-registration indicated that 8.2% of the examined veterinary medication samples were labeled as being of poor quality, and ~12 (1.3%) of the examined products had flaws in their appearance, packaging, or labeling (25). Veterinary drugs and biological products produced, imported, distributed, and used in the country are not effectively regulated and managed in terms of quality, safety, and efficacy (2, 22).

Currently, due to the prevalence of animal diseases, VPs are widely used. However, in Africa, including Ethiopia, the VP sector, especially veterinary supplies warehouse management practices, is a neglected research area, except for some fragmented studies that show the presence of poor handling of veterinary drugs (4, 5, 24). Besides, the researcher had the opportunity to visit a few veterinary drug warehouses and noticed and understood that warehouse management practices lacked attention even though the products were expensive, unique, and dealt with animal life.

Implementing good veterinary supplies, particularly through WM, in the context of veterinary health facilities served as inspiration for addressing such types of issues and maintaining the overall veterinary pharmaceutical supply chain operations, which are crucial to improving veterinary healthcare services through the provision of quality VPs. Hence, the current study was conducted to assess the veterinary supplies warehouse management practices in relation to stock management, storage conditions, warehousing activities (receiving, storing, and issuing), human and material resources, and identifying the challenges encountered linked to the fundamental veterinary supplies warehouse operations at government district veterinary clinics and private veterinary drug wholesalers in four selected zones and Bahir Dar city of the Amhara region, Ethiopia.

## Methods and materials

### Study area and period

The study was conducted in four selected zones and Bahir Dar administrative city in the Amhara region of Ethiopia from April 1, 2022, to January 15, 2023. The Amhara region is located in the north-western part of Addis Ababa, the capital city of Ethiopia. The study area covers the northern and western parts of the region. Administratively, there are 12 zones in the Amhara region. For this study, Bahir Dar City (the capital city of the Amhara region) was purposefully selected because most veterinary drug wholesalers are located in Bahir Dar City. The four zones, namely south Gondar, central Gondar, west Gondar, and west Gojam zones were selected based on the inclusion criteria of political stability and security concern at the time of data collection and veterinary health services coverage. In the study area, according to the 2022 report acquired from the Veterinary Drug and Feed Administration Control Authority (VDFACA) of the Amhara regional branch and the Amhara regional state livestock and fisheries resource development office, there are 66 facilities (14 private veterinary drug wholesalers and 52 government district veterinary clinics) serving to care for over 23 million livestock. Most veterinary drug wholesalers are found in the region's capital city, while others are scattered at the zonal level, and government veterinary clinics are at the district/woreda level.

### Study design

A facility-based descriptive cross-sectional study design complemented by qualitative research approaches was conducted. The study mostly used a quantitative approach to produce numerical data, whereas a qualitative approach was used to explore the challenges faced in the catchment area and strengthen the quantitative data. The survey was done at veterinary health facilities using self-administered questionnaires, observational checklists, and face-to-face interviews with key informants (KI).

### Source and study population

#### Source population

The source population included all government veterinary clinics, all private veterinary drug wholesalers, all veterinary health professionals working with veterinary drugs in the warehouse and storage areas, and those who had a position related to pharmaceutical supply chain management in the four specified zones and Bahir Dar administrative city of Amhara regional state, Ethiopia.

#### Study population

Selected district government veterinary clinics and private veterinary drug wholesalers in the selected four zones and Bahir Dar city of the Amhara region were assessed to collect the necessary data. The government district veterinary clinic's veterinary drug and input supply officer, veterinary drug store personnel and veterinary drug dispenser, veterinary drug wholesale owners, wholesaler technical managers, wholesaler assistant storekeepers, district livestock and fishery resource development heads, and animal health department coordinators were all contacted and invited to participate in this study.

#### Inclusion and exclusion criteria

District veterinary clinics, veterinary drug wholesalers, and employees working with veterinary drugs and had positions related to veterinary drug handling and management practices for at least the last 6 months during data collection participated in the study. Zones that suffered from political instability and security concerns during data collection were excluded. Government animal health posts located at the kebele level, private veterinary clinics, and private retail outlets were also excluded as they did not have a permit to hold veterinary pharmaceutical stock.

### Sample size determination and sampling techniques

#### Sample size determination

The formula developed by Cochran in 1963 for calculating sample sizes when the proportion is the parameter of research was applied, and a 90% confidence level with a 10% margin of error was used. Using this formula and assuming a 10% non-respondent rate, 37 health facilities were selected as a study sample from a refined population of 66 facilities (government district veterinary clinics and private veterinary drug wholesalers) in the study area. The quantitative and qualitative data were obtained from 29 government district veterinary clinics and eight private veterinary drug wholesalers (Supplementary material 1).

The general formula was calculated using Equation (1):


(1) $$\begin{array}{lll} no & = & \frac{\left(z2*p*q\right)}{e2} \end{array}$$

$$\begin{array}{lll} no & = & \frac{\left(1.64\right)2*0.5\left(1-0.5\right)}{\left(0.1\right)2}=67. \end{array}$$

Where, no is the sample size required, *Z* is the *Z* value (1.64 for a 90% confidence level), and p is the estimated prevalence of the indicator. The product of [p] and [q] is maximized when *p* = 0.5. Therefore, when the prevalence is unknown, 0.5 should be used, and e2 = the 10% margin of error used in estimating the prevalence, e2 = 0.1. However, the sample size (n_0_) was adjusted to n: This adjustment can substantially reduce the necessary sample size for small populations and is also called the population correction factor (26) (Equation 2):


(2) $$\begin{array}{l} n=\frac{no}{\left[1+\left\{\frac{no-1}{N}\right\}\right]} \end{array}$$

Where, n = the adjusted new sample size; N = the population size; n_0_ = the sample size obtained from the general formula.


$$\begin{array}{l} \text{n }=\;\frac{67}{1\;+\left[\left(67\;-\;1\right)/66\right]} \end{array}$$

n = 33.5–34 facilities, with a 10% non-response rate, 37 facilities.

Accordingly, 37 veterinary health facilities were selected proportionally from the four zones and Bahir Dar city, and 75 participants working in the selected veterinary health facilities participated. The selection was purposefully made based on their direct involvement in pharmaceutical warehouse management activities and their position in the facilities. Practically, during data collection, some facilities did not have veterinary drug and input supply employees, others did not have drug store and control employees, and a few others did not have a drug dispenser. Due to the small size of the target population in the selected facilities, all 75 professionals participated in this study. Key informants for qualitative data collection were determined based on the viewpoints of different researchers and the principle of data saturation (27, 28). The KIs were selected from the 37 veterinary health facilities. Their positions, being decision-makers and having information on the issues of veterinary supply warehouse management practices, were considered during the selection of KIs. Accordingly, the districts' livestock and fishery resource development heads, animal health department coordinators, private veterinary drug wholesale owners, and veterinary drug wholesaler technical managers working from the selected facilities were invited.

#### Sampling techniques

In the Amhara region, there are 12 zones, so it was difficult to address all district veterinary clinics and private veterinary drug wholesalers in these zones. Therefore, for general representation of the study site, the four zones and Bahir Dar administrative city were selected based on the inclusion criteria and because of their high density of veterinary health facilities and service coverage. Veterinary clinics and drug wholesalers in the four zones and Bahir Dar city were completely enumerated and listed. Then, the health facilities were stratified according to the types of facilities, such as government district veterinary clinics or private veterinary drug wholesalers. Finally, the number of veterinary health facilities included in the sample from each stratum was determined using a proportional size allocation technique (28). The study was conducted at 37 facilities, of which 29 were government district veterinary clinics, and eight were private veterinary drug wholesalers, selected by a simple random sampling technique (Supplementary material 2).

Regarding study participants, district veterinary clinic drug store personnel, district veterinary clinic drug store manager, district veterinary drug input and supply officer (from the selected government district veterinary clinics), wholesaler veterinary drug assistant storekeeper, and wholesaler veterinary drug technical manager (from private veterinary health facilities) were invited to fill out the self-administered structured questionnaire. For key informants, the district livestock and fishery resource development heads, animal health department coordinators, private veterinary drug wholesale owners, and veterinary drug wholesaler technical managers (from the selected private veterinary health facilities) were invited to participate in the interview. The selection of the KI was based on their position as decision-makers and because they are familiar with pharmaceutical supply chain information and related activities. Moreover, they also have information about the challenges and related factors in the veterinary supplies warehouse management practice.

### Data collection tools and procedures

To collect the primary data, structured self-administered five-level Likert scale questionnaires [that were rated from strongly disagree to strongly agree where 1 = strongly disagree, 2 = disagree, 3 = neutral, 4 = agree, and 5 = strongly agree], observational checklists, and a semi-structured interview guide [adopted from standard criteria from the Logistic Indicator Assessment Tool (LIAT) developed by the USAID Deliver Project and data collection tools] from various related articles were referred for adoption and customized to the local context (11, 24, 29–31).

Two veterinary pharmacists and the principal investigators were allocated and participated as data collectors for quantitative and qualitative data. Quantitative data used to assess veterinary pharmaceutical stock management-related practices was collected using structured, self-administered questionnaires. The five-level Likert scale questionnaires were used to assess warehousing activities and human and material resource management practices at the selected facilities, and 75 participants filled out the questionnaire. The veterinary supplies storage conditions of the facilities were evaluated using checklists through direct physical observation (Supplementary material 3), and a semi-structured, open-ended interview guide was used to collect the qualitative data through face-to-face interviews.

The district veterinary clinics veterinary drug and input supply officer, veterinary drug store and control personnel, drug dispensers, veterinary drug wholesalers technical managers, and assistant storekeepers from the sampled facilities participated in filling out the self-administered questionnaire. The qualitative data was collected through face-to-face interviews with KIs using the prepared interview guide. Interviews were conducted with district livestock and fishery resource development heads, district animal health department coordinators, private veterinary drug wholesaler owners, and wholesaler technical managers. Interview guides were prepared in English and then translated into Amharic, the working and local language in the study area. The principal investigator interviewed the KIs in depth for an average of 30 min. Notes were taken, and KI responses were also audio-taped.

### Data quality assurance

The study questionnaire and interview guide were derived from a standard tool and developed after reviewing previously studied related research (11, 24, 30). To maintain the quality of the data and to encourage the meaningful participation of the respondents, the layout of the questionnaires was kept clear and very simple. Prior to data collection, the principal investigator provided training to data collectors on data collection procedures and the significance of the study. Before being entered into the Statistical Packages for Social Science (SPSS) and MS Excel, the collected data was carefully checked for accuracy, cleaned for completeness, consistency, omissions, and irregularities., Every day, during data collection, the misunderstood questions were elaborated accordingly. To ensure the reliability of self-administered questionnaires and the respondent's understanding of the questions, the questionnaire was pretested with 5% of the total sample size of the study, which is not included in the study area.

A scale reliability test was conducted for Likert scale items and reliability analysis; Cronbach's alpha was calculated using SPSS version 26. If the Cronbach's alpha coefficient is close to 1.0, then there is greater internal consistency in the items, and a value >0.700 is considered very acceptable for SCM activities (72). For this study, the value of Cronbach's alpha (Table 1) for the Likert scale questionnaire is >0.70.

**Table 1:** Summary of the reliability analysis test.

**No**	**Variables**	**Result of Cronbach's alpha**
1	Receiving activities	0.709
2	Storing activities	0.825
3	Issuing activities	0.778
4	Human and material resource management	0.733

For qualitative data, the probing and flexible questions and interview guide were initially prepared in English and then translated into Amharic by the principal investigator after consulting with people with good command of the two languages. Data collection was undertaken by the researcher, and the interviews were transcribed each day after the interview. Missing ideas and any need for clarification were addressed throughout the process. For data consistency and completeness, all Amharic transcripts were cross-checked with the oral discourse. After repeated reading of the filled-out notes and careful listening to the audio records, coding, and recoding of the contents were done with peer review. The principal investigator also used the reflexivity method to improve the quality of data collection, which enabled better probing, fewer assumptions, the avoidance of premature interpretation, and an accentuated sense of curiosity during the interview (32).

### Data analysis and interpretation

The collected primary data was used to show the magnitude of stock management, storage conditions of the facilities, warehousing activities of the facilities using the target variables (receiving, storing, and issuing), and human and material resource management practices at a facility level. The quantitative data was coded and entered into SPSS version 26 and Microsoft Excel 2010 for analysis. Descriptive statistics (frequency, percentage, mean, and standard deviations) were computed, and summary results were presented using tables, graphs, and charts. The qualitative data obtained from the in-depth interview was analyzed and summarized using a thematic approach (33). The grand mean and standard deviation (SD) were used to interpret the Likert scale data gathered to assess the warehousing activities of the target variables (receiving, storing, and issuing) and the human and material resource management practices at a facility level. Each warehousing activity (receiving, storing, and issuing) was assessed using four items, and human and material resource management practices were assessed using seven items. The grand mean and SD of the target variables were computed from the respective items (Table 7). The interval range of the 5-likert scale (Table 2) was calculated according to the principle of the grouped data frequency distribution formula (34). The mean of their response scores for each variable represented their level of satisfaction with pharmaceutical warehousing activities and human and material resource management practices, whereas the SD represented their deviation from the central value (35, 36).

**Table 2:** The interval range of Likert scale questionnaires used for this study.

**Level**	**Scale**	**Interval length**	**Interval range**
Strongly Disagree	1	0.8	1–1.8
Disagree	2	0.8	1.81–2.60
Neutral	3	0.8	2.61–3.4
Agree	4	0.8	3.41–4.2
Strongly Agree	5	0.8	4.21–5

Source: Computed using grouped data frequency distribution formula.

The results of the computed mean were then leveled as “strongly disagreeing” if a variable with a mean score fell in the interval of 1–1.8, “disagreeing” if the score fell in the interval of 1.81–2.60, “neutral” if the score fell in the interval of 2.61–3.4, “agreeing” if the score fell in the interval of 3.41–4.2, and “strongly agreeing” if the score fell in the interval of 4.21–5. An SD of > 0.9 implies a significant difference in the target variable among respondents (37, 38).

To interpret the results of the mean and SD easily and clearly, the scales were reassigned as follows, and the verbal interpretation was made based on the recommendations of previous researchers (37–39) (Table 3).

**Table 3:** Verbal interpretation of the scale.

**Interval range**	**Level**	**Interpretation**
1-1.8	Strongly disagree	Very dissatisfied
1.81-2.60	Disagree	Dissatisfied
2.61-3.4	Neutral	Moderately satisfied
3.41-4.2	Agree	Satisfied
4.21-5	Strongly Agree	Very satisfied

Source: Hailiu (39).

The percentage of storage conditions was calculated as the average number of “yes” responses in the checklist and the number of standard storage conditions in the checklist (40) using Equation (3).


(5) $$\begin{array}{l} \;\%\text{ storage condition}\\ =\frac{\text{The number of yes response in the checklist}}{\text{Number of standard storage conditions and checklist}} \end{array}$$

The management of storage conditions associated with veterinary pharmaceutical products was also classified as “poor” and “good” store management. The interpretation was made based on the recommendation from previous research and the principles of pharmaceutical warehouse operations management of the Ethiopian Pharmaceutical Fund Supply Agency (PFSA), now known as the Ethiopian Pharmaceutical Supply Services (EPSS), published in 2015 (11). Based on this, pharmaceutical warehouses or facilities that fulfilled at least 80% of the criteria for good storage conditions were considered acceptable and have good storage conditions, whereas those that fulfilled <80% were considered poor storage conditions (40).

For the qualitative data, the principal investigator performed face-to-face, in-depth interviews to explore the challenges faced in veterinary pharmaceutical warehouse management practices. The investigator transcribed the audio recordings of in-depth interviews and discussions verbatim. Textual notes and audio-recorded data were repeatedly read and listened to. Audio recordings and notes were translated into English. The thematic analysis technique was used to analyze the data collected from the KIs as per the approach and steps recommended by Braun and Clarke (41). By doing so, the investigator became familiar with the textual notes and audio recordings and began taking notes accordingly. Then, the data was coded and written up using MS Word. The coded data was organized to search for themes and subthemes. After that, similar subthemes were grouped, named, and described thematically. Thematic contents were formulated, and a master list of themes was developed based on the research questions and conceptual framework. Finally, the report was produced using an exploratory approach and triangulated with the quantitative result.

## Results

### The background information of veterinary health facilities and respondents' profile

**Figure 1:**
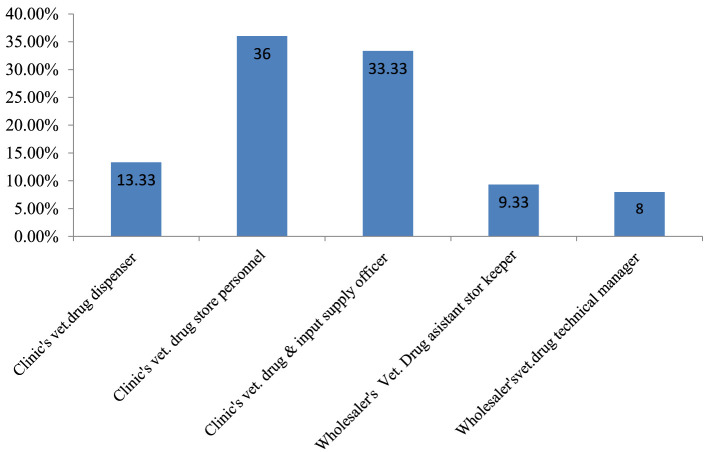
Professional designations of the respondents from district government veterinary clinics and private veterinary drug wholesalers.

To assess pharmaceutical warehouse management practices and their challenges, 37 veterinary health facilities−29 (78.4%) district veterinary clinics and 8 (21.6%) private veterinary drug wholesalers—were invited. All 37 facilities participated with a 100% response rate. Among the respondents, the majority (27; 36%) were district veterinary clinic drug store personnel, and 6 (8%) were veterinary drug wholesaler technical managers (Figure 1).

**Figure 2:**
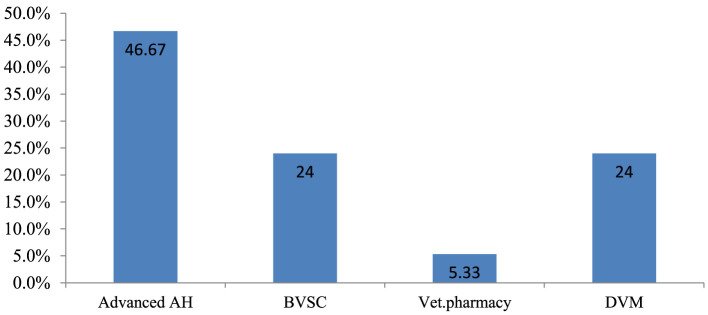
Professional qualifications of the respondents. AH, Animal health; BVSC, Bachelor of Veterinary Science; Vet, Veterinary; DVM, Doctor of Veterinary Medicine.

The majority of respondents (35; 46.7%) were qualified in advanced animal health, and 4 (5.3%) had veterinary pharmacy professional qualifications (Figure 2). Furthermore, 36 (48%) of the respondents held degrees, 35 (46.7%) held diplomas, and 4 (5.3%) had obtained MSc level of education. In terms of work experience, 42 (56%) had 3–6 years, 18 (24%) had >7 years, and 15 (20%) had 0–2 years of experience.

Advanced animal health: animal health professionals who took advanced animal health qualification courses at the college level for 3 years and were certified.

Bachelor of Veterinary Science: Veterinary professionals who took animal health qualification courses at the university level for 3 years and were certified.

Veterinary Pharmacy: Professionals who have been certified by taking full veterinary pharmacy qualification courses for 4 years at the university level.

Doctor of Veterinary Medicine: a veterinary professional who took animal health qualification courses at the university level for 6 years and was certified.

### Assessment of veterinary drug warehouse management practices of the facilities

#### Veterinary pharmaceutical stock management-related practices of the facility

All the facilities managed both veterinary drugs (medicines) and other equipment and supplies used in veterinary healthcare services. The majority of the facilities (26; 70.3%) used mixed-type drug arrangement methods, while 8 (21.6%) used pharmacological drug arrangement methods. Bin cards and system software/electronic data interchange technology were not used in any of the surveyed facilities. Of the surveyed facilities, in 23 (62.2%), pharmaceutical information was handled manually or on paper, and 4 (10.8%) utilized mixed-based information handling methods. More than half of the facilities (59.5%) also reported that they did not have a written manual or standard operating procedure (SOP) to manage warehouse practices. The majority of facilities did not dispose of expired products on time (32; 86.5%) and did not have documented policies and guidelines (31; 83.8%) for the management of veterinary drug waste (Table 4).

**Table 4:** Summary of stock management practices at the facility.

**Description of variables**		**Frequency (percent)**
Types of veterinary pharmaceutical products managed at the facilities	Veterinary drugs and equipment	37 (100%)
Pharmaceutical product arrangement methods	Mixed type	26 (70.3%
	Pharmacologic-therapeutic order	8 (21.6%)
	Dosage form	3 (8.1%)
	Alphabetical order	0 (0%)
Pharmaceutical information handling methods in the facility	Manual/paper-based	23 (62.2%)
	Computerized	10 (27%)
	Mixed-based	4 (10.8%)
Stock recording forms used to manage products	Stock cards	37 (100 %)
	Bin cards	0 (0%)
	Self-inspection and audit	37(100 %)
Disposal of expired products	Facilities not disposing of expired products on time	32 (86.5%)
System-related issues	Facilities do not have document policies/guidelines for pharmaceutical waste management	31 (83.8%)
	Facilities do not have SOPs	22 (59.5%)
	Facilities use system/software	0 (0%)
	Facilities are not supervised and do not receive feedback from regulatory bodies	16 (43.2%)

### Assessment of storage conditions by facility type

#### Storage conditions of district government veterinary clinics

The storage areas of 29 governmental veterinary clinics were assessed through physical observation using 20 criteria. The study found that the majority (27; 93.1%) of the facilities were protected from direct sunlight−19 (65.5%) stores had separate storage and dispensing areas. However, none of the stores had fire safety equipment or wall thermometers. Only 14 (48.3%) facilities had separate storage areas for expired and damaged products and a very limited number of stores (20.7%) had pallets and shelves. In only a few stores were products stacked at least 20 cm away from the walls (17.2%), 10 cm off the floor (10.3%), and on racks over 2.5 m in length (17.2%). Overall, the average performance of district government veterinary clinics that met the criteria for acceptable storage conditions was 48.3% (Supplementary material 4).

**Figure 3:**
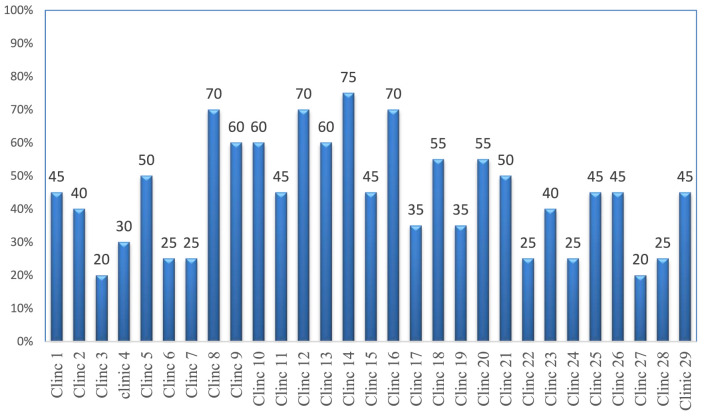
Performance of storage conditions at government district veterinary clinics.

Of the 29 surveyed government district veterinary health clinics, no facility met the criteria for good storage conditions, and seven had a storage condition performance of 25% or below (Figure 3).

#### Storage conditions in private veterinary drug wholesalers

**Figure 4:**
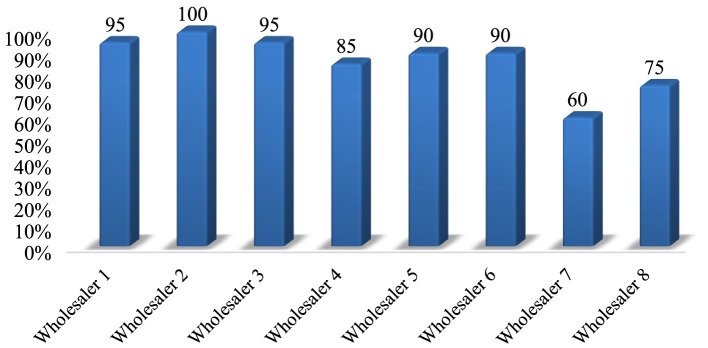
Performance of storage conditions at private veterinary drug wholesalers.

The stores of 8 private veterinary drug wholesalers were assessed through physical observation using 20 criteria. The study found that products were protected from direct sunlight in all stores. In all the stores visited, palettes and shelves were accessible, and dispensing and storage areas were separated. The majority (75%) of the facilities had separate storage rooms for damaged and expired goods, and during the physical inspection, all stores looked free from harmful insects and rodents. Only four facilities (50%) had separate and specialized storage areas for flammable products and chemicals. Products were stacked at least 20 cm away from walls, 10 cm from the floor, and on racks that were 2.5 m in length in the majority of stores (87.5%) inspected. Overall, the average performance of private veterinary drug wholesalers that complied with the acceptable storage criteria was 86.25% (Supplementary material 5). Of the eight private drug wholesalers surveyed, six met the criteria for good storage conditions with an average percentage >80% (Figure 4).

**Figure 5:**
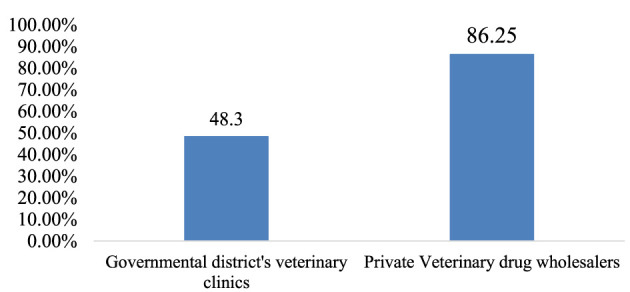
Average adherence to acceptable storage conditions by facility type.

The overall adherence to storage conditions in the district government veterinary clinics and private veterinary drug wholesalers was, on average, 48.3 and 86.25%, respectively (Figure 5).

### Assessment of veterinary pharmaceutical warehousing activities

The warehousing activities (receiving, storing, and issuing) of the surveyed facilities were analyzed using descriptive statistics, and the grand mean and standard deviation of the target variables were computed (Table 5).

**Table 5:** The grand mean and standard deviation of the target variables.

**Target variables**	**Descriptive statistics**	

	**Grand mean**	**Std. deviation**
Receiving activities of the facilities	3.31	0.64
Storing activities of the facilities	2.53	0.89
Issuing activities of the facilities	4.07	0.44
Human and material resources of the facilities	2.40	0.61

#### Receiving activities

The majority of respondents had a “neutral” response to their facility's pharmaceutical receiving activities, with a mean value of 3.31 and an SD of 0.64. The individual response for each item in the receiving activities is shown in Table 6.

**Table 6:** Respondent's perception of each item in the receiving activities of the facility.

**Receiving activities**	**Response categories in frequency (** ***n*** **) and percentage (%)**					**Result of descriptive statistics**		
**Items/questions**	**Strongly disagree**	**Disagree**	**Neutral**	**Agree**	**Strongly agree**	**Mean**	**SD**	**Total**
	***n*** **(%)**	***n*** **(%)**	***n*** **(%)**	***n*** **(%)**	***n*** **(%)**			
In our drug warehouse, there is a pre-notification area for incoming pharmaceutical products.	4 (5.3)	18 (24)	13 (17.3)	37 (49.3)	3 (4)	3.23	1.03	75
There are procedures for the cross-checking and identification of documents and products received.	0	5 (6.7)	2 (2.7)	60 (80.0)	8 (10.6)	3.95	0.63	75
There are procedures for the notification of discrepancies to the suppliers for the returning and receiving of products.	0	9 (12)	15 (20)	43 (57.3)	8 (10.7)	3.67	0.83	75
In our warehouse, the receiving space is safe for the movement of product-handling equipment	6 (8)	46 (61.3)	13 (17.3)	6 (8)	4 (5.3)	2.41	0.95	75
**Grand mean and SD**						3.31	0.64	75

#### Storing activities

The majority of respondents were found to “disagree” with their facility's pharmaceutical storing activities, with a mean value of 2.53 and an SD of 0.89. The individual response to each item in the storing activities is shown in Table 7.

**Table 7:** Respondent's perception of each item in the storing activities of the facility.

**Storing activities**	**Response categories in frequency (** ***n*** **) and percentage (%)**					**Descriptive statistics**		
**Items/question**	**Strongly disagree**	**Disagree**	**Neutral**	**Agree**	**Strongly agree**	**Mean**	**SD**	**Total**
	***n*** **(%)**	***n*** **(%)**	***n*** **(%)**	***n*** **(%)**	***n*** **(%)**			
In our warehouse, there are adequate storage areas to store the inspected products.	15 (20)	40 (53.3)	6 (8)	10 (13.3)	4 (5.3)	2.31	1.10	75
In our warehouse, most of the drugs are arranged in the storage area as clearly identified with their categories.	3 (4)	30 (40)	12 (16)	24 (32)	6 (8)	3.00	1.10	75
In our warehouse, the location of the stored products is clearly recorded and traceable.	8 (10.7)	44 (58.7)	5 (6.7)	13 (17.3)	5 (6.7)	2.51	1.11	75
In our warehouse, products are stored according to the manufacturer's storage specification at all times.	17 (22.7)	35 (46.7)	9 (12.0)	10 (13.3)	4 (5.3)	2.32	1.13	75
**Grand mean and SD**						2.53	0.89	75

#### Issuing activity

The study found that the majority of respondents agreed on their facility's pharmaceutical issuing activities, with a mean value of 4.07 and an SD of 0.44. The individual response to each item in the issuing activities is shown in Table 8.

**Table 8:** Respondent's perception of each item in the issuing activities of the facility.

**Issuing activities**	**Response categories in frequency (** ***n*** **) and percentage (%)**					**Descriptive statistics**		
**Items/questions**	**Strongly disagree**	**Disagree**	**Neutral**	**Agree**	**Strongly agree**	**Mean**	**SD**	**Total**
	***n*** **(%)**	***n*** **(%)**	***n*** **(%)**	***n*** **(%)**	***n*** **(%)**			
Most of the time, products are picked based on the printed order-picking format.	0	0	0	37 (49.3)	38 (50.7)	4.51	0.50	75
In our warehouse, most of the time, products are picked in the order of FEFO/first expiry first out principle.	0	0	0	52 (69.3)	23 (30.7)	4.31	0.46	75
Warehouse workers always update records when goods are issued from their storage areas.	0	1(1.3)	0	52 (69.3)	22 (29.3)	4.27	0.53	75
In our storage area, there are enough space for product packing, labeling, and dispatching.	0	12 (16)	36 (48)	26 (34.7)	1(1.3)	3.21	0.72	75
**Grand mean and SD**						4.07	0.44	75

### Assessment of human and materials resource management practices of the facilities

The current study found that the majority of respondents “disagreed” with the facilities' human and material resource management practices, with a mean value of 2.40 and an SD of 0.61 (Supplementary material 6). The socio-demographic data collected for this study indicated that the majority (71; 94.7%) of employees had non-veterinary pharmacy professional qualifications, and only 4 (5.3%) were veterinary pharmacy professionals (Figure 2). The study also found that 43 (57.3%) participants had not received or participated in any on-the-job training sessions (Table 9).

**Table 9:** Summary of training history of respondents.

	**Have you ever received on-the-job training?**		

		**Frequency (** ***n*** **)**	**Percent (%)**
Valid	No	43	57.3%
	Yes	32	42.7%
	Total	75	100.0%

**Figure 6:**
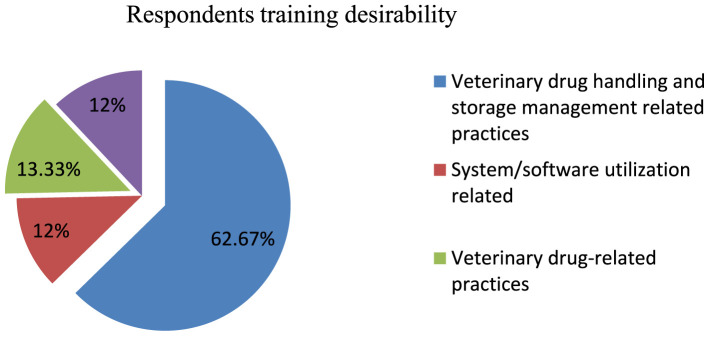
Training desirability among respondents.

When the respondents were asked about training, all of them indicated their desire to take training in the future, and they pointed out the types of training they required (Figure 6).

### Results of qualitative data

In this study, qualitative data was collected using a face-to-face interview to identify and explore the challenges faced by government district veterinary clinics and private veterinary drug wholesalers in managing their veterinary supplies warehouses.

### Socio-demographic characteristics of key informants

**Figure 7:**
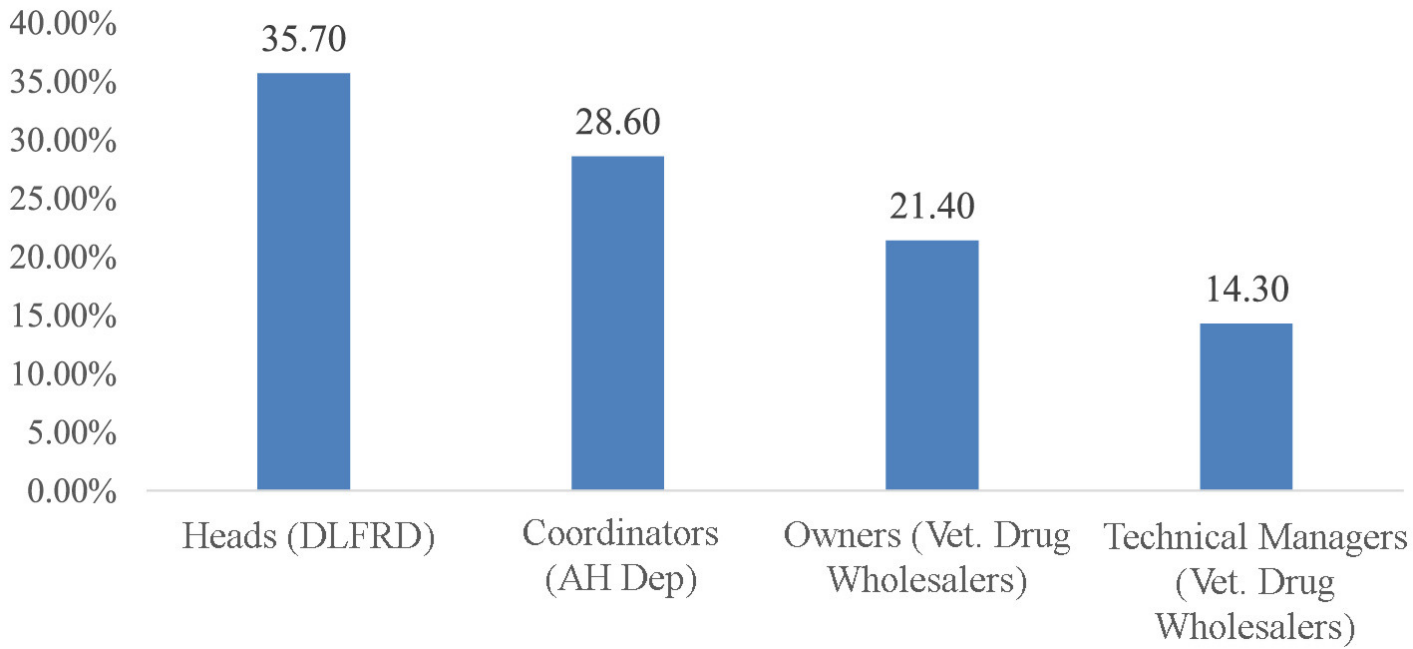
Socio-demographic characteristics of key informants. DLFRD, District Livestock and Fishery Resource Development; AH dept, Animal Health Department; Vet, veterinary.

A total of 14 KIs were interviewed for this study. The majority of them (5; 35.7%) were District Livestock and Fishery Resource Development heads, 4 (28.6%) were Animal Health Department coordinators, and the remaining were from private veterinary drug wholesalers (Figure 7). Concerning their educational qualifications, 5 (35.7%) had a doctorate in Veterinary Medicine, 5 (35.7%) had a bachelor's degree in Veterinary Science, 2 (21.4%) had a diploma in Advanced Animal Health, and 2 (21.4%) had a degree in Veterinary Pharmacy.

### The assessment of the challenges linked to the veterinary pharmaceutical warehouse management practices

As perceived by the KIs, the surveyed facilities face enormous challenges related to veterinary pharmaceutical warehouse management practices. Based on the characteristics of the data, the challenges were divided into three major thematic areas. These were infrastructure challenges, human and material resource challenges, and challenges related to the sale and purchase of veterinary drugs.

### Theme one: challenges associated with infrastructure

This theme focuses on challenges raised regarding the warehouse and storage areas of veterinary pharmaceutical products. Key informants reported that the lack of adequate storage space is a challenge in almost all facilities. Most KIs mentioned that “the layout of our pharmaceutical warehouse is not designed based on the standard of drug storage and lacks storage space to accommodate all the stock appropriately” (heads, District Livestock and Fishery Resource Development and coordinators, Animal Health Department).

This statement was supported by one other KI who said:

The main challenge in our district is the inadequacy of drug storerooms. As you have seen, our drugstore is very narrow and old, and we do not have enough pallets and shelves. Our drug store is not free from leakage of water, dust, or direct sunlight. We have no warehouse built for this purpose. To store drugs, we assign empty buildings or offices. It is not as per the standards (Coordinator, District Animal Health, BVSc, 3 years of experience).

Another KI stated:

Due to a shortage of adequate storage space, we are forced to store different products, like flammable chemicals, laboratory reagents, expired products, and even non-functional equipment, together with unexpired pharmaceutical products, in the same storage area. This makes it impossible to track the product accurately. But we have no option (Head, District Livestock And Fishery Resource Development, MSc, 5 years of experience).

One more KI emphasized:

The drug storage area is our major challenge. During a meeting at the district and regional level, I wrote a letter and forwarded questions to district leaders and to other higher governmental bodies. But still, this problem is not solved. This is due to budget constraints and the lack of awareness and attention of district leaders and other higher governmental bodies in this sector. Some even think that veterinary drugs do not need special warehouses and storage areas because they consider veterinary drugs to be like other materials (Head, District Livestock And Fishery Resource Development, DVM, 3 years of experience).

On infrastructure, almost all respondents from the private drug wholesalers emphasized that issues related to building rent are their major challenges. “There is a time-to-time rent cost increment, and it isn't easy to search for standard buildings that fit with the directives of VDFACA. So, it creates a big challenge for our business” (Owners and Technical Managers, Private Wholesalers).

### Theme two: challenges associated with human and material resources

#### Human resource

Most key informants mentioned that the shortage of qualified staff to perform activities like drug storage, issuing, and dispensing is a challenge. A KI remarked, “In our woreda, most of the district clinic drug stores and dispensers are run by non-pharmacy professionals.” One KI stated:

This is the only district clinic in this woreda, and we have 37 clinics at the kebele level. Conversely, we have only one district drug store control person. He performs many activities. He works as an accountant by giving receipts to customers. At the same time, he dispenses drugs. He also works as a drug store employee, issuing drugs to professionals coming from each kebele. There is a high workload among the available professionals. There was no compensation for work overload, and there were no educational opportunities. So, how can we be effective in drug handling and management? (District livestock and fishery resource development heads and animal health department coordinators, Coordinator, Animal Health Department, BVSc, 7 years of experience).

This KI also confirmed that “there is no job description used for district drug dispensary and drug store personnel.”

#### Training related challenges

All KI interviewed from district clinics mentioned that their main challenge in pharmaceutical warehouse management was the lack of equipment and materials such as fire extinguishers, ventilation, wall thermometers, computers, cold chain materials/ice boxes, and vehicles used for drug transportation (heads, District Livestock and Fishery Resource Development and coordinators, Animal Health Department).

This statement was emphasized by another KI:

Our district is grouped under the desert area, so the drug store needs ventilation and a wall thermometer to control the temperature daily, but as you see, our wall thermometer and the ventilation have not been functional for the last 2 months. Non-functional equipment is not timely renewed (Coordinator, District Animal Health Department, DVM, 5 years of experience).

Yet another KI added:

We have no vehicle for transporting drugs from our supplier and distributing drugs to our kebele clinics. Drugs are transported in public vehicles like Bajaj, motorcycles, or even by human carriage. This exposes drugs to external factors like direct sunlight, which reduces the quality of the drug (Head, District Livestock and Fishery Resource Development, BVSc, 9 years of experience).

All the KI mentioned that they need system software and standardized manuals. One KI admitted that these manuals “facilitate our warehousing activities, but still no one uses them.” Another KI added, “our activities are not electronic. This is due to budget constraints and a lack of computers. Our staff is not also trained in this regard.” (heads, District Livestock and Fishery Resource Development and coordinators, Animal Health Department).

### Theme three: challenges associated with the sale or purchase of veterinary drugs

KIs were interviewed about the challenges faced in the sale and purchase of pharmaceuticals, and all highlighted that availability, affordability, financial resources, and regulatory-related challenges were their major issues.

#### Availability and affordability of veterinary drugs

According to the KIs, the availability and ability to obtain essential veterinary drugs at an affordable cost are their biggest challenges. In particular, the KIs from drug wholesalers indicated that there was an inadequate supply of veterinary drugs. For instance, one KI said, “Importers are not able to supply the drugs needed by our customers. Even now, it is difficult to afford the drugs available on the market. The lack of availability of essential veterinary drugs is our major challenge in our commercial endeavors.” Another KI added: “As an example, when we see pen-strep, it has not been available on the market for the last 2 months, and its cost has increased from 170 to 650 ETB per vial” (Technical Manager and Owner, Veterinary pharmaceutical warehouse, 2 years of experience).

All the KIs from the district clinics also emphasized this statement by saying that:

Drug availability is our major challenge. We have had no pen-strep for the last 4 months. This is very essential for treating the majority of animal diseases. The Amhara region veterinary drug and input supply agency is the main supplier for all districts in the region, but the agency cannot supply as per our requisition.

One KI strongly highlighted,

We frequently face a shortage of animal drugs. We do not get some items on the market because of the current shortage of hard currency in our country, and due to this, it is difficult to deliver full services to the community (heads, District Livestock and Fishery Resource Development and coordinators, Animal Health Department).

#### Regulatory-related challenges

Most KIs from district clinics mentioned that “government regulatory bodies like VDFACA and other regional and zonal agricultural and livestock offices do not support us in the fulfillment of pharmaceutical warehouses except for some irregular training they deliver.” Another KI added by saying, “We expect more from VDFACA in addition to facilitating training to realize the quality of veterinary drugs. But still, their effort in this regard is very low” (heads, District Livestock and Fishery Resource Development and coordinators, Animal Health Department).

All the KIs from the drug wholesalers emphasized that “The lack of a regulatory and service chain between regional and federal regulatory bodies of VDFACA is a challenge. Making contracts with our employees (technical managers and assistant storekeepers) and even renewing our license requires a trip to the central FDFACA.” On this issue, all the respondents appeared quite emotional and exasperated and asked, “Why is the Amhara regional branch of the VDFACA unable to perform these tasks? This creates a big challenge for our services.”

Most of the KIs also highlighted that “Disposing of expired and unusable pharmaceutical products is our challenge. There are teams or committee members organized from different sectors, but the team is not working actively. We don't have policies and guidelines to manage these waste products” (heads, District Livestock and Fishery Resource Development and coordinators, Animal Health Department). One KI from the government district clinic emphasized, “I have worked in this district for over 15 years, including the last 5 years as the district livestock resource coordinator. In our drug store, there are many expired vaccines, drugs, and chemicals that were in our clinics and collected from kebele clinics starting 10 years ago and are still lying in our store. We always write a letter to the district managers, but they are still not committed to implementing this” (Coordinator, Animal Health Department, BVSc, 16 years of experience).

#### Budget-related challenges

Most KIs from the district clinics mentioned that: “Inadequate allocations of budgets are the major challenge for us to fulfill regulations relating to premises, including infrastructure, buildings, and human and material resources, which are essential for achieving good practices in pharmaceutical warehouse management and providing basic service at the facility level.” A majority of them support the argument that “even within the sector, budget allocation is not fair since the livestock sector has merged with agriculture. Most of the budget is allocated for the agricultural and livestock production wings rather than the maintenance of livestock health” (heads, District Livestock and Fishery Resource Development and coordinators, Animal Health Department).

## Discussion

To maintain the quality and efficacy of pharmaceutical products, good warehouse management is central and requires attention among other pharmaceutical supply chain activities. To provide effective health services, whether in animal or human health aspects, a pharmaceutical warehouse and store should be properly installed, and drugs should be properly managed and handled (11, 14). Without proper pharmaceutical storage management, the entire healthcare system will fail. In the livestock sector, high-income countries have established systems for assessing and monitoring the quality of veterinary products available in the market and at service delivery sites, whereas most low- and middle-income countries still struggle to monitor the proper use of veterinary medications (4).

Literature suggests that pharmaceuticals should be clearly organized and arranged with each zone of the store to make it much easier for store personnel to control stock, take periodic stock inventories, pick orders, and time will not be wasted (42, 43). The current study found that in the majority (26; 70.3%) of surveyed facilities, mixed-type veterinary drug arrangement methods were used. This finding is higher than the finding of the study conducted on the pharmaceutical storage of public health centers at North Shoa Zone, which showed that in 29.3% of facilities, products were arranged in mixed types. The observed difference could be due to the availability of adequate storage space and sufficient shelves and pallets in the medical health sector. In that study, shelves were sufficiently available in 27 (65.9%) facilities out of 41 health facilities (44), whereas the availability of pallets and shelves in the current study was only about 60.35% on average from the surveyed 37 facilities.

Implementing automated systems is essential for managing warehouse operations and enables warehouse managers to complete their responsibilities more quickly, precisely, affordably, and flexibly (3, 41, 42). The present study found that none of the facilities had system software or electronic data interchange technology, 23 (62.2%) facilities handled pharmaceutical-related information, and everything was paper-based. This contradicted the findings of a study conducted at private medical drug wholesalers in Gondar, Ethiopia, which showed that 80% of the surveyed facilities' pharmaceutical warehouse management practices used the Professional Electronic Data System (PEDS) (11).

This difference might be due to the type of health facilities studied and the fact that the amount of stock managed in the medical pharmacy store may be huge, making it difficult to manage that huge stock manually. The non-use of system software in veterinary health facilities may be due to financial constraints on computer access and a lack of trained professionals. The quantitative result of this study indicated that the activities being carried out in the surveyed facilities were manual and paper-based. This was also supported by the qualitative result, in which budget-related constraints, computer access, and a lack of trained staff were the major challenges to automating their warehousing practices. Therefore, these findings establish that veterinary pharmaceutical stock arrangements and pharmaceutical-related information are not handled in an organized way.

Even if using a bin card is a time-consuming and laborious task, implementing this professional tool enables store managers to accomplish their activities in a rapid, effective, and cost-effective manner (11). However, this study revealed that bin cards were not utilized in any of the surveyed facilities. This finding contradicts the results of a study conducted on inventory management of laboratory commodities in Gambela regional state and Jimma zone, Southwest Ethiopia, which found that utilization of bin cards was 58.8 and 69.9%, respectively (45, 46). Additionally, the result is also contrary to the findings of the study conducted in public health centers on pharmaceutical store management practices in Addis Ababa and the North Shoa Zone, which indicated that bin card utilization was 48.9 and 54%, respectively (30, 44).

The quantitative result of the current study showed that the performance of bin card utilization in the surveyed veterinary health facilities was very low. The qualitative result also supported this finding with KIs noting the presence of a professional awareness gap on the use of bin cards; low ownership and attention given by higher managerial units; less commitment of the district veterinary drug and input supply officer; a lack of trained and qualified store personnel; and a lack of supportive supervision by regulatory bodies and district leaders. This indicated poor implementation of veterinary supplies stock-keeping practices in the surveyed veterinary health facilities.

The standard operational procedure simplifies the warehouse's operations by providing specific step-by-step instructions for each activity and ensuring the quality of the activities performed in the warehouse uses the same measurable standards every time (47). However, the present study found that 22 (59.5%) facilities had no standardized written manual. It was lower than the results of the study conducted in public health centers and hospitals in Dessie Town, Ethiopia, which showed that 80% of the facilities had standard guidelines for managing commodities in their stores (48). The observed difference might be due to differences in study facilities. The finding of this study was to deduce if in the majority of veterinary health facilities, warehouse practices are performed randomly or as per standards.

Scientific evidence recommends that expired or damaged stocks should be immediately removed from the usable inventory and sent to a separate place according to the established guidelines. This is because pharmaceutical waste could be dangerous and may pollute the environment habituated by the general public or wildlife, or even be diverted to the marketplace for illegal resale (49, 50). However, the present study found that the majority (32; 86.5%) of surveyed veterinary health facilities did not dispose of expired products promptly, and 31 (83.8%) facilities did not have documented policies and guidelines for managing veterinary drug waste. The disposal practices found in the current study were poor compared to the study conducted in the North Shoa Zone, which showed 63.4% of health facilities disposed of pharmaceutical waste. In a study conducted in Addis Ababa, 66.7% of the health centers disposed of pharmaceutical waste within a year, and the availability of waste disposal documents was 100% (44, 51). The observed difference could be due to differences in study settings, as currently, the human health sector is implementing an integrated pharmaceutical logistics system throughout health facilities and using the pharmaceutical waste rate as one of the key performance indicators for pharmaceutical logistics. According to the quantitative result of the current study, the expired and waste product disposal practices in the veterinary health sector were poor. This was also strengthened by the results obtained from the face-to-face interview, as the majority of KIs stated that most facilities do not have policies or guidelines to manage waste products. Some government district clinics have not disposed of expired products for the past 10 years.

### Storage conditions of the facilities

Storage conditions are regarded as the cornerstone of warehouse management practices. Any defect in the storage area may result in obsolescence, deterioration, spoilage, pilferage, or breakage of stock due to excessive overstocking. Furthermore, the poisonous degradation of products can be hazardous to humans and the environment (30, 52). The present study revealed that the average percentage of storage conditions in government district veterinary clinics and private veterinary drug wholesalers is 48.3 and 86.25%, respectively. This observed difference in storage performance between governmental and private entities could be because private veterinary drug wholesalers might be subject to inspections by government regulatory bodies. Furthermore, they also face strict control for the layout and fulfillment of the warehouse premises before their license is issued. According to this study's finding, the government district veterinary clinics did not meet the criteria for acceptable storage conditions, which was below the acceptable range (80%).

This finding is consistent with the study results conducted on assessing pharmaceutical store management practices in public hospitals in Addis Ababa, which showed an average adherence to proper storage conditions at 47.1% (30). The similarity of the findings could be that both were governmental facilities, so regulatory bodies and management units may not pay attention. However, the result was lower than the results of the study conducted on inventory management for laboratory commodities from health facilities in Gambela regional state and Jimma zone, Ethiopia, which indicated that the overall adherence to the criteria for proper storage conditions was 68.2 and 70.6%, respectively (45, 53). The current study generally indicated the storage conditions in governmental district veterinary clinics were poor and below the acceptable limit (≥80%). This could be due to a lack of adequate storage space. The qualitative result also supports this because most of the KIs invited for interviews stated that the main challenge in their districts was the inadequacy of drug storerooms and the lack of standardized design and layout.

On the other hand, private veterinary drug wholesalers met the criteria for acceptable storage conditions, with an overall performance of 86.25%, which is considerably good (≥80%) (40). The percentage of storage conditions in private veterinary pharmaceutical wholesalers found in this study was higher than those of a study conducted on pharmaceutical warehouse management practices among private medical pharmaceutical wholesalers in Gondar, Ethiopia, which found that the facilities' storage performance was 68.75%. The difference could be due to the commitment of the concerned regulatory bodies to inspecting, controlling, providing feedback, and supervising the facilities.

### Warehousing activities of the facilities

Receiving, storing, and issuing/shipping goods are the key operational tasks carried out in the warehouse, and proper practice of all the tasks is vital to warehouse management (54). Scientific studies suggest that warehouse management practices may differ across different sectors. It depends on various variables, such as material turnover and demand specifications, the type of materials used, the organizational unit's operational scope, and its size (55). Researchers recommend that if the tasks of receiving in a warehouse are not operated properly, they make up roughly 10% of operating expenditures in any distribution center (56). Regarding the receiving activities, the present study found that the majority of respondents had “neutral” responses to the performance of pharmaceutical receiving activities at the facility level, with a computed mean value of 3.31 that falls within the range of 2.61–3.4 and an SD of 0.64. From the analysis, it can be deduced that the current pharmaceutical receiving activity of the veterinary health facilities, which includes the availability of a pre-notification area for incoming pharmaceutical products, procedures for the cross-checking and identification of the documents and products received, procedures for the notification of discrepancies to the suppliers for the returning and receiving of products, and the safety of the receiving space for the movement of products handling equipment, is moderately satisfied. The SD of 0.64 indicates that there were no extremes in respondents' positive or negative scores. As stated in the literature, receiving activities should get strict attention, as they make up roughly 10% of operating expenditures in any distribution center. However, the qualitative result of this study indicated that governmental bodies did not pay attention to the sector, especially for the fulfillment of infrastructure and storage premises (56).

Regarding the storing activities, the majority of respondents “disagreed” with their facility's performance of pharmaceutical storing operations, with a computed mean value of 2.53 that falls within the range of 1.81–2.60 and an SD of 0.89. This indicated the current pharmaceutical storage activity of the veterinary health facilities, which includes the availability of adequate storage areas to store the inspected products, the arrangement of drugs in the storage area as clearly identified with their categories, the availability of clearly recorded and traceable locations for storing products, and the fact that products are stored according to the manufacturer's storage specifications at all times, were unsatisfactory. The standard deviation of 0.89 indicates that there are no extremes in respondents' positive or negative scores. Researchers suggest that storing activities cost ~15% of warehouse operating costs (56). However, the qualitative findings of this study show that the storage activities of the facilities are not based on standards.

Regarding issuing activities, this study found that the majority of respondents were “in agreement” with a computed mean value of 4.07 that falls within the range of 3.4–4.2 and an SD of 0.44 for the performance of the facility's pharmaceutical issuing activities. From the analysis, it can be deduced that the pharmaceutical issuing activity of the veterinary health facilities in terms of products is picked based on the printed order picking format; products are picked in the order of the FEFO principle; records are updated when goods are issued from their storage areas; and the availability of enough areas for product packing, labeling, and dispatching is “satisfactory.” An SD of 0.44 indicates that the majority of respondents had similar reflections.

### Human and material resources management practices

In pharmaceutical warehouse management practices, the personnel who work there and handle the materials have a direct role in managing the stock and all other warehouse operations (53). Researchers also suggested that effective pharmaceutical warehouse management is determined by the professional's qualification level, training and capacity building, and the accessibility of sufficient material and equipment (which are crucial because they guard against future harm to the workers and the warehouse) (11, 54). The present study found that the availability of qualified and sufficient numbers of staff to manage warehouse operations and the availability of equipment and materials used for facilitating warehouse activities at the surveyed facilities were unsatisfactory, with a computed mean value of 2.40 falling within the range of 1.81–2.60, and an SD of 0.61. This finding establishes that the human and material resource management practices, which comprise the availability of a sufficient number of staff, awareness of staff on veterinary pharmaceutical warehouse management principles, availability of job descriptions for their respected duties, availability of sufficient materials and equipment like personal protective materials, store safety materials like fire extinguishers, ladders and pallet jacks, hand trucks, etc. to facilitate warehouse activities, and the delivery of timely maintenance support and replacement for the equipment in the warehouses when it is not working satisfactorily. The SD of 0.61 indicates that the majority of respondents had similar reflections.

Evidence also suggests that the level of exposure to pharmaceutical warehouse management practices and other related supply chain activities is different for different professionals. Medicine storage is one of the most important responsibilities that can be best handled by a pharmacist (57). Accordingly, the veterinary pharmacy professional has direct exposure to the related tasks compared to non-pharmacy animal health professionals. The present study found that most of the veterinary pharmaceutical warehouse management activities were performed by non-pharmacy professionals (71, 94.7%); on the other hand, the involvement of veterinary pharmacy professionals in pharmaceutical warehouse practice was only about 4 (5.3%). This is in line with the result of the study conducted on the assessment of inventory and store management practices of pharmaceuticals in public health centers and hospitals in Dessie Town, Ethiopia, which showed only two institutions (20%) completely controlled and operated their stores by pharmacists (48). However, this was lower than the result of the study conducted in India, where 60% of the health centers were operated by pharmacists (58). This observed difference could be due to the difference in the study population.

The observed difference might also be due to the availability of educated human resources and the absence of job descriptions. As stated in the literature, a review conducted on veterinary drug management, handling, utilization, resistance, and side effects confirmed that low educational levels and a lack of graduates in veterinary medicine who are aware of pharmaceutical warehouse management were the major problems in veterinary drug handling and management (14). The absence of a job description for veterinary pharmacy professionals was supported by a qualitative result, as key informants confirmed that there is no job description used for district drug dispensaries and drug store personnel.

Different scholars suggest that employing qualified warehouse personnel and providing necessary training is crucial in improving the productivity of warehousing operations (10, 53). However, the present study revealed that more than half (57.3%) of the study participants had not received on-the-job training in veterinary drug management, handling, and other related activities. The findings are somewhat consistent with a study carried out on the veterinary drug supply chain in Uganda, which found that nearly 90% of drug retailers and veterinary drug practitioners did not receive specialized training in veterinary medicine handling and storage management. The findings are also consistent with another study conducted on the assessment of veterinary drug handling, management, and supply chain in Ethiopia's Afar Pastoral Region, which found that ~63.9% of respondents lacked sufficient knowledge on safe handling and management of veterinary drugs (21, 24).

## Strengths and limitations of the study

This study was the first in the country to assess the status of veterinary pharmaceutical warehouse management practices and will serve as a baseline for future research. Furthermore, the strength of this study was that it used both quantitative and qualitative approaches in assessing existing practices and the challenges of veterinary supplies warehouse management practices. However, due to insufficient previous studies conducted in veterinary pharmaceutical warehouse management practices in the study area and abroad, it was difficult to compare the results with those conducted in similar settings. Besides, due to time constraints, geographic distance, and financial limitations, this study did not cover all the facilities available in the study area.

## Conclusion and recommendations

This study revealed that most of the surveyed facilities in the study area did not prioritize the management practices of veterinary supply warehouses. The study specifically found that the warehouse management practices at government district veterinary clinics and private drug wholesalers were unsatisfactory. This was evident because 23 (59.5%) facilities lacked standard operating procedures for warehouse activities, and no veterinary health facilities utilized bin cards and system software. Furthermore, the majority of facilities (32; 86.5%) did not have guidelines for drug disposal and failed to dispose of expired drugs on time. The storage conditions at government district veterinary clinics were poor, with 48.3% meeting below the minimum requirements for good storage conditions. In contrast, the storage conditions at private veterinary drug wholesalers were good, with 86.25% meeting the necessary standards.

On warehousing activities, the storing activities and human and material resource management practices of the surveyed facilities were not satisfactory. Key informants highlighted several challenges that hindered effective veterinary supplies warehouse management, such as inadequate infrastructure, lack of qualified and trained staff, insufficient storage safety and security equipment, issues with pharmaceutical product availability and affordability, weak regulatory framework, and budget constraints at the facility level. Overall, the study found that warehouse management practices in the surveyed facilities were significantly poor. To enhance the management practices of veterinary pharmaceutical warehouses, various entities, including the District Veterinary Health Service offices, zonal Agricultural and Veterinary Health Services offices, Amhara Region Livestock and Fishery Resource Development office, Veterinary Drug and Feed Administration Control Authority, and veterinary professionals must make concerted efforts.

## Data availability statement

The original contributions presented in the study are included in the article/Supplementary material, further inquiries can be directed to the corresponding author.

## Ethics statement

The study was conducted after obtaining approval and clearance letters from the University of Gondar, School of Pharmacy with Ref No. S/A/P/67/2014. To collect data from the selected facilities letters of permission were obtained from the Amhara region livestock and fishery resource development office and the Ethiopian veterinary drug and feed control administration authority, Amhara regional branch. The participants were asked and provided their oral informed consent to participate in this study.

## Author contributions

AW: Conceptualization, Data curation, Formal analysis, Investigation, Methodology, Software, Validation, Writing—original draft. TS: Project administration, Supervision, Visualization, Writing—review & editing. AE: Project administration, Supervision, Visualization, Writing—review & editing. YT: Data curation, Validation, Visualizations, Supervision, Writing—review & editing. BW: Project administration, Supervision, Visualization, Writing—review & editing.
