# Supplementary material for: Assessment of veterinary pharmaceutical warehouse management practices and its associated challenges in four selected zones and Bahir Dar city of Amhara regional state, Ethiopia
Source: Front Vet Sci. 2024 May 7;11:1336660. doi: 10.3389/fvets.2024.1336660 (PMC11107088; doi:10.3389/fvets.2024.1336660)
Supplement: Supplementary file 1 [file Table_1.docx]

Supplementary Table 1: Selected facilities for the study and number of veterinary health facilities available in the study area (N = 66)

| **Selected Study area** | **Total number of facility in the study area** | |  | **Number of selected facilities for this study by facility type** | | |
| --- | --- | --- | --- | --- | --- | --- |
|  | **veterinary Drug wholesalers** | **Governmental Veterinary clinics** |  | **Drug wholesalers** |  | **Veterinary clinics** |
|  |  |  |  |  | |  |
| 1. Bahir Dar administrative City | 10 | 0 | 6 | | | 0 |
|  |  |  |  | | |  |
| 1. South Gondar zone | 1 | 15 |  | 1 | | 8 |
|  | 3 |  |  |  | |  |
| 1. West Gojam zone |  | 17 |  | 1 | | 10 |
|  |  |  |  |  | |  |
| 1. Central Gondar zone | 0 | 15 |  | - | | 8 |
|  |  |  |  |  | |  |
| 1. West Gondar zone | 0 | 5 |  | - | | 3 |
| **Total number of facilities selected** |  | | **37** | | | |
