# Supplementary material for: Assessment of veterinary pharmaceutical warehouse management practices and its associated challenges in four selected zones and Bahir Dar city of Amhara regional state, Ethiopia
Source: Front Vet Sci. 2024 May 7;11:1336660. doi: 10.3389/fvets.2024.1336660 (PMC11107088; doi:10.3389/fvets.2024.1336660)
Supplement: Supplementary file 2 [file Table_2.docx]

Supplementary Table 2. Checklists used to assess the physical storage condition of veterinary health facilities (governmental district veterinary clinics and private veterinary drug wholesalers).

|  | **Descriptions of the statements Responses category**  **Yes No** |  | |
| --- | --- | --- | --- |
|  |  |  |  |
|  |  |  |  |
| 1 | Availability of separate storage and dispensing area |  |  |
| 2 | Availability of palates and shelf’s in the storage area |  |  |
| 3 | Identification labels, manufacturing dates and expiry dates are visible |  |  |
| 4 | Cartons and products are in good condition not crushed due to mishandling |  |  |
| 5 | Cartons and products are protected from water and humidity |  |  |
| 6 | Products are protected from direct sunlight |  |  |
| 7 | The storage area is visually free from harmful insects and rodents |  |  |
| 8 | Availability of separate storage area for expired and damaged products from usable  Products |  |  |
| 9 | The current space are organized and sufficient for existing products |  |  |
| 10 | The roof is maintained in good conditions to avoid sunlight and water penetration |  |  |
| 11 | Room is maintained in good condition( all trash removed, clean and organized shelves and boxes) |  |  |
| 12 | Fire safety equipment and wall thermometer are available |  |  |
| 13 | Flammable products and chemicals are stored separately in specialized area |  |  |
| 14 | Products are stacked at least 20 cm away from the walls and other stacks |  |  |
| 15 | Products are stacked at least 10 cm off the floor |  |  |
| 16 | Products are stacked with at least 2.5 m length of the rack |  |  |
| 17 | Availability of cold chain maintenance equipment’s like refrigerators and ice box in the store |  |  |
| 18 | Availability of enough space for the movements of good handling equipment and warehouse workers |  |  |
| 19 | Store room have placement of door/window/ grills or iron bar for security |  |  |
| 20 | Availability of office table, chair and toilet |  |  |
