# Supplementary material for: Assessment of veterinary pharmaceutical warehouse management practices and its associated challenges in four selected zones and Bahir Dar city of Amhara regional state, Ethiopia
Source: Front Vet Sci. 2024 May 7;11:1336660. doi: 10.3389/fvets.2024.1336660 (PMC11107088; doi:10.3389/fvets.2024.1336660)
Supplement: Supplementary file 3 [file Table_3.docx]

Supplementary Table 3: Summary of storage condition of district veterinary clinics (n=29)

| No | **Descriptions of the statements** | **Responses category**  **frequency n [%]** | |
| --- | --- | --- | --- |
|  |  | Yes | No |
|  |  |  |  |
| 1 | Availability of separate storage and dispensing area | 19 (65.5) | 10 (34.5) |
| 2 | Availability of palates and shelf’s in the storage area | 6 (20.7) | 23 (79.3) |
| 3 | Identification labels, manufacturing dates and expiry dates are visible | 14 (48.3) | 15 (51.7) |
| 4 | Cartons and products are in good condition not crushed due to mishandling | 16 (55.8) | 13 (44.2) |
| 5 | Cartons and products are protected from water and humidity | 25 (86.2) | 4 (13.8) |
| 6 | Products are protected from direct sunlight | 27 (93.1) | 2 (6.9) |
| 7 | The storage area is visually free from harmful insects and rodents | 17 (58.6) | 12 (41.4) |
| 8 | Availability of separate storage area for expired and damaged products from usable Products | 14 (48.3) | 15 (51.7) |
| 9 | The current space are organized and sufficient for existing products | 4 (13.8) | 25 (86.2) |
| 10 | The roof is maintained in good conditions to avoid sunlight and water penetration | 19 (65.5) | 10 (34.5) |
| 11 | Room is maintained in good condition( all trash removed, clean and organized shelves and boxes) | 13 (44.8) | 16 (55.2) |
| 12 | Fire safety equipment and wall thermometer are available | 0 | 29 (100%) |
| 13 | Flammable products and chemicals are stored separately in specialized area | 2 (6.9) | 27 (93.1) |
| 14 | Products are stacked at least 20 cm away from the walls and other stacks | 5 (17.2) | 24 (82.8) |
| 15 | Products are stacked at least 10 cm off the floor | 3 (10.3) | 26 (89.7) |
| 16 | Products are stacked with at least 2.5 m length of the rack | 5 (17.2) | 24 (82.8) |
| 17 | Availability of cold chain maintenance equipment’s like refrigerators and ice box in the store | 25 ( 86.2) | 4 (13.8) |
| 18 | Availability of enough space for the movements of good handling equipment and warehouse workers | 21 (72.4) | 8 (27.6) |
| 19 | Store room have placement of door/window/ grills or iron bar for security | 25 (86.2) | 25 (13.8) |
| 20 | Availability of office table, chair and toilet | 20 (69.0) | 9 (31.0) |
|  | **Average** | **48.3** |  |
