# Supplementary material for: Assessment of veterinary pharmaceutical warehouse management practices and its associated challenges in four selected zones and Bahir Dar city of Amhara regional state, Ethiopia
Source: Front Vet Sci. 2024 May 7;11:1336660. doi: 10.3389/fvets.2024.1336660 (PMC11107088; doi:10.3389/fvets.2024.1336660)
Supplement: Supplementary file 4 [file Table_4.docx]

**Supplementary Table 4:** Summary of physical storage condition of the private veterinary drug wholesalers (n=8)

| No | **Descriptions of the statements** | **Responses category**  **Frequency n [ %]** | |
| --- | --- | --- | --- |
|  |  | **Yes** | **No** |
| 1 | Availability of separate storage and dispensing area | 8 (100%) | 0 |
| 2 | Availability of palates and shelf’s in the storage area | 8 (100%) | 0 |
| 3 | Identification labels, manufacturing dates and expiry dates are visible | 5 (62.5%) | 3(37.5%) |
| 4 | Cartons and products are in good condition not crushed due to mishandling | 7 (87.5%) | 1(12.5%) |
| 5 | Cartons and products are protected from water and humidity | 8 (100%) | 0 |
| 6 | Products are protected from direct sunlight | 8 (100%) | 0 |
| 7 | The storage area is visually free from harmful insects and rodents | 6 (75%) | 2(25%) |
| 8 | Availability of separate storage area for expired and damaged products from usable Products | 6 (75%) | 2(25%) |
| 9 | The current space are organized and sufficient for existing products | 5 (62.5%) | 3(37.5%) |
| 10 | The roof is maintained in good conditions to avoid sunlight and water penetration | 8 (100%) | 0 |
| 11 | Room is maintained in good condition ( all trash removed, clean and organized shelves and boxes) | 6 (75%) | 2(25%) |
| 12 | Fire safety equipment and wall thermometer are available | 7(87.5%) | 1(12.5%) |
| 13 | Flammable products and chemicals are stored separately in specialized area | 4 (50%) | 4 (50%) |
| 14 | Products are stacked at least 20 cm away from the walls and other stacks | 7 (87.5%) | 1(12.5%) |
| 15 | Products are stacked at least 10 cm off the floor | 8 (100%) | 0 |
| 16 | Products are stacked with at least 2.5 m length of the rack | 8 (100%) | 0 |
| 17 | Availability of cold chain maintenance equipment’s like refrigerators and ice box in the store | 8 (100%) | 0 |
| 18 | Availability of enough space for the movements of good handling equipment and warehouse workers | 8 (100%) | 0 |
| 19 | Store room have placement of door/window/ grills or iron bar for security | 5 (62.5) | 3(37.5%) |
| 20 | Availability of office table, chair and toilet | 8 (100%) | 0 |
|  | **Average** | 86.25 |  |
