# Supplementary material for: Assessment of veterinary pharmaceutical warehouse management practices and its associated challenges in four selected zones and Bahir Dar city of Amhara regional state, Ethiopia
Source: Front Vet Sci. 2024 May 7;11:1336660. doi: 10.3389/fvets.2024.1336660 (PMC11107088; doi:10.3389/fvets.2024.1336660)
Supplement: Supplementary file 5 [file Table_5.docx]

**Supplementary Table 5.** Respondents' perceptions for human and material management practices of the facility

| **Human and material resource management practices** | | **Response categories in (frequency and percentage)** | | | | | | **Descriptive statistics** | | | |
| --- | --- | --- | --- | --- | --- | --- | --- | --- | --- | --- | --- |
|  |  | **SD**  n (%) | **D**  n (%) | | **N**  n (%) | **A**  n (%) | **SA**  n (%) | **Mean** | | **SD** | **Total** |
|  |  |  |  | |  |  |  |  | |  |  |
|  | There are sufficient number of staffs to run the activities in the warehouse | 11 (14.7) | | 41 (54.7) | 7 (9.3) | 13(17.3) | 3(4.0) | 2.41 | | 1.07 | 75 |
| The staff members are aware of pharmaceutical warehouse management principles | | 5 (6.7) | | 34(45.4) | 22(29.3) | 13(17.3) | 1(1.3) | | 2.61 | 0.89 | 75 |
| The warehouse personnel have job description for their respected duties | | 7(9.3) | | 32(42.7) | 15(20.0) | 21(28.0) | 0 | | 2.67 | 0.99 | 75 |
| There are sufficient personal protective materials like gown , masks and gloves for the staff working in the store | | 3 (4.0) | | 21(28.0) | 18(24.0) | 31(41.3) | 2(2.7) | | 3.11 | 0.98 | 75 |
| In our warehouse safety materials like fire extinguishers and alarm for unexpected incidents are available sufficiently | | 28 (37.3) | | 33(44.0) | 2(2.7) | 8(10.7) | 4(5.3) | | 2.03 | 1.15 | 75 |
| There are sufficient materials and equipment’s like ladder, pallet jacks, hand truck etc. to facilitate the warehouse activities | | 35 (46.7) | | 37(49.3) | 2(2.7) | 1(1.3) | 0 | | 1.59 | 2.40 | 75 |
| In our warehouse there is maintenance support and replacement of equipment in the warehouse when they are not working  **Grand mean and SD** | | 16 (21.3) | | 34(45.3) | 5(6.7) | 19 (25.3) | 1(1.3) | | 0.617 | 1.127 | 75 |
|  |  |  | |  | | | | | **2.40** | **.61** | 75 |
